# Monitoring Local Earthquakes in Central Italy Using 4C Single Station Data

**DOI:** 10.3390/s21134297

**Published:** 2021-06-23

**Authors:** Andreino Simonelli, Matteo Desiderio, Aladino Govoni, Gaetano De Luca, Angela Di Virgilio

**Affiliations:** 1 Section of Pisa, Istituto Nazionale di Fisica Nucleare, 56123 Pisa, Italy; matteo.desiderio.20@ucl.ac.uk (M.D.); angela.divirgilio@pi.infn.it (A.D.V.); 2 Istituto Nazionale di Geofisica e Vulcanologia, 00143 Roma, Italy; aladino.govoni@ingv.it (A.G.); gaetano.deluca@ingv.it (G.D.L.)

**Keywords:** earthquakes location, rotational measurements, local earthquakes

## Abstract

In this study, performed on a set of twenty-two earthquakes that occurred in central Italy between 2019 and 2020, we will explore the possibility to locate the hypocenter of local events by using a ring laser gyroscope observing the vertical ground rotation and a standard broadband seismometer. A picking algorithm exploiting the four components (4C) polarization properties of the wavefield is used to identify the first shear onset transversely polarized (SH). The wavefield direction is estimated by correlation between the vertical rotation rate and the transverse acceleration. The picked times for Pg and Sg onsets are compared to the ones obtained after manual revision on the GIGS station seismometer. The results are compared with the location provided by the national monitoring service of the INGV.

## 1. Introduction

The possibility to measure the rotation of the ground induced by seismic waves or seismic noise opened the way to new studies in seismology. The need for observing this long time known degree of freedom was stated a long time ago by [1]. The sensitivity of modern large ring laser gyroscopes like [2,3] is indeed sufficient to detect events at tele-seismic distances as well as local earthquakes, where present. The operative range is comparable to modern broadband seismometers.

The instruments used for this study are the Gingerino ring laser gyroscope [4] and a broadband seismometer trillium 240 co-located on top of the granite frame of the gyroscope. The standard seismometer is sensitive to translations of the ground, and the gyroscope provides us in the case of Gingerino, the vertical rotation rate. The Gingerino gyroscope has been developed as a pathfinder for general relativity tests [5,6]. This ensemble of instruments constitutes a four-component (4C) seismic station, and they are available on EIDA under the station name GIGS of the INGV network [7]. The Gingerino instrument right after his commissioning permitted several seismological studies from distant earthquakes [8,9] to storm generated noise [10] and finally local earthquakes in [11] i.e., the topic of this paper. The theory underneath rotations in seismology is largely explored in literature [12,13] and new processing studies are available [14,15] as well as new instruments [16]. For this reason, we avoid retreating to the mathematical derivation of fundamental equations for data interpretation. We need instead to support the advances in this branch of science with new data and analysis. A seismic station where rotational observables are present is comparable to a seismometer array in terms of the scientific results that we can obtain from it. In fact, an estimation of the direction of the seismic wave field and phase velocity of Rayleigh waves or Love waves, depending on the orientation and the number of rotational components observed, is possible and already reported in literature. To solve the problem of earthquake location, we need to extract from our data a distance or, in circular coordinates, a radius, and an orientation i.e., an angle that is the direction of the incoming wave field: the back-azimuth (hereinafter BAZ). We have shown in different works that we are able to identify the BAZ, and we are left with the problem of identifying the distance. To solve this problem, we will rely on the time difference between p to s arrivals; the details of the analysis will be explained in the following section. A learning process is used to convert time differences into distances by comparing the location provided by the INGV.

## 2. The Rotational Sensor: Gingerino

**Figure 1:**
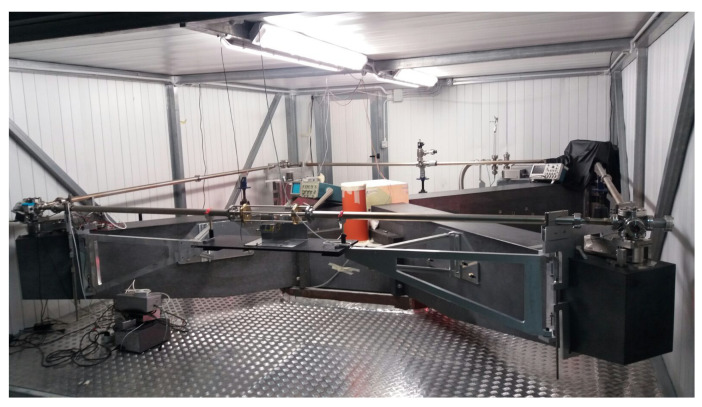
The Gingerino active ring laser gyroscope inside the Gran Sasso national laboratory of the INFN.

An active Ring Laser Gyroscope (RLG) exploits the Sagnac effect to measure the rotation rate [17]. In this instrument, on the contrary, propagating laser beams generated inside a ring resonator undergo a frequency split δf If the ring laser optical cavity is rotating. For a horizontal RLG, located at colatitude θ, the splitting δf induced by the Earth’s rotation rate $\Omega_{E}$, is expressed in the function of the cavity area *A*, perimeter *P*, and laser-wavelength λ:

(1) $$\delta f=\frac{4A\Omega_{E}}{\lambda P}\cos\left(\theta +\delta \phi_{NS}\right)\cos\delta \phi_{EW},$$

where $\delta \phi_{NS}$ and $\delta \phi_{EW}$ are the tilt angle in the North–South and in the East–West directions. GINGERino RLG operates on the HE:Ne red line at 633 nm. The square optical cavity of 3.6 m on a side is defined by four spherical mirrors with a 4 m radius of curvature. According to Equation (1), given the horizontal orientation of the RLG and at the latitude of LNGS, the frequency splitting due to earth rotation is 280.4Hz. The optical path of the laser beams is enclosed inside the cavity in a steel vacuum chamber, composed by four mirror chambers connected by vacuum pipes. The alignment of the spherical mirrors can be tuned by means of micrometric tip-tilt systems acting on the mirror chambers’ orientation. Transmission losses of all four mirrors and the presence of eight optical windows installed on each corner permit extracting from every corner a portion (in terms of power) of the two counter propagating laser beams. A Pyrex capillary with an internal diameter of 5.5 mm is installed in the middle of one side. On the capillary, two electrodes driven with radio frequency excitation are bolted. The RF excitation creates a plasma discharge inside the optical cavity where the laser effect occurs. In an optical cavity, many modes can exist, i.e., transverse and longitudinal ones. The capillary itself forces the laser to operate on single transverse mode (${\mathrm{TEM}}_{00}$), while single longitudinal mode operation is obtained by keeping the laser excitation close to the threshold. The four mirror chambers are tightly attached to a cross structure made of granite (african black), composed of a central bulky block of three tons, and four lightened arms, each weighting ≈800kg (see Figure 1).

The granite structure is connected to a reinforced concrete block anchored to the underneath bedrock. Given Equation (1), it is easily understandable by simple error propagation that we want both area and perimeter as steady as possible to minimize the error on frequency estimation, a way to achieve that is to avoid thermal expansion. There are two ways to do that: control the temperature both passively and actively and install the optical cavity on a zero expansion coefficient material. The African black granite has been a good choice because it can be machined with high precision and has a low thermal expansion coefficient ($6.5\times 10^{-6}$/°C). The thermal stability of the cave is very good but at the price of a very high humidity; this is why the whole installation is now protected by a large anechoic box. Infrared lamps are used to increase the temperature inside the box and reduce the relative humidity from more than 90% down to about 50–60%. The Gingerino RLG has been running since 2016 at a temperature around 13 °C with a stability better than 0.1 °C with a duty cycle better than 90% On top of the central part of the granite frame, we installed a seismometer provided by the INGV Trillium 240. The seismometer and the RLG are digitized by two six-channel nanometrics centaur instruments, and the raw data are collected in MSEED format. By means of seedlink connection, the row data of the RLG are sent to a computer where the seiscomp environment is running, and a specific plugin is dedicated to the data demodulation whose output is a value in cycles per second representing the beat note frequency. By using Equation (1), it is then possible to obtain a scalar value that permits to convert the beat note frequency in rotation rate. This value is included in the dataless file of the RLG, and the instrumental deconvolution can be performed via the usual “remove sensitivity” routine of Obspy. Both the seismometer’s channels from the Trillium and the rotational ones demodulated by the seiscomp plugin are streamed to the EIDA knot of the INGV [7] with a station name of GIGS, and the rotational channels are streamed under the ‘*JZ’ name at 2, 20 and 100 Hz of sampling rate. The data coverage of the rotational channel starts from February 2020. The data used in this paper are pre processed using the method described above that is bound in the Obspy package.

## 3. Method

The location of earthquakes is a problem typically solved with many stations by various national earthquakes monitoring agencies; however, it is also possible to locate a seismic source using a single three-component seismometer [18]. The method is described in many works—for example, [19]; however, we believe that the use of an additional rotational observable constrains much more the solution of the problem since a four-components seismic station can be considered as a point-like array [20,21]. The benefits of using the rotation rate observable will permit solving the π ambiguity of the classic BAZ estimation via wave polarization (the only method possible with only a seismometer) and will possibly allow a better s waves picking [22]. The unique location of our setup, in the middle of a very active seismic region and a very well instrumented area, permits a comparison between the location provided by the INGV and the one obtained with our 4C seismic station. The availability of a single 4C station will give a great logistic advantage in poorly instrumented areas and open the possibility to perform a real-time processing of the data at the station and then provide a preliminary estimation of the epicenter location, avoiding the transmission of the entire data stream.

Locating an earthquake on one single station requires the estimation of a distance and of the back azimuth, i.e., the angle from station to epicenter measured anticlockwise:

(2) $$D=\Delta t_{ps}\frac{v_{p}v_{s}}{v_{p}-v_{s}}$$

**Figure 2:**
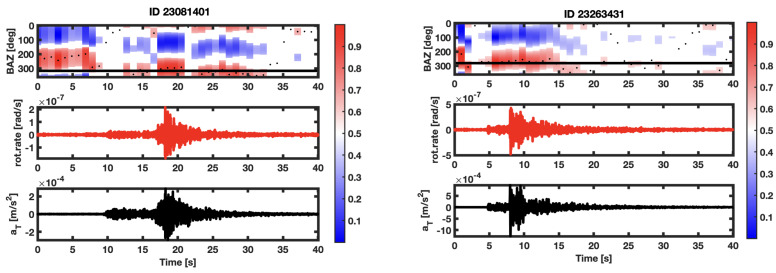
Example of BAZanalysis for two selected events.

The estimation of event BAZ with a 4C seismic station is a very well proven technique by many studies not only for our setup [11], but also for other stations [3] and portable sensors [16]. We recall here the basic equation that connects cinematically the observable vertical rotation rate to transverse acceleration in Equation (3):

(3) $$\omega_{z}=-\frac{\ddot{x}}{C_{Lg}}$$

where $\ddot{x}$ is the acceleration measured by a seismometer caused by a transverse polarized wave propagating in a generic direction named y, $\omega_{z}$ in the vertical rotation rate and $c_{Lg}$ is the phase velocity of a crustal transversely polarized phase. Maximizing the zero lag correlation between the two cinematic observable of Equation (3) permits finding the back-azimuth of the incoming wave field provided that we have two horizontal components from the co-located seismometer. We numerically rotate the North and East components and, step by step, we compare it to the vertical rotation rate. The angle at which the maximum value occurs is the one that we are confident to be the real BAZ. The π ambiguity is then solved at the origin by cinematic constraints from Equation (3). It can be obtained by different processing techniques, and all of them must converge to the same result; in our case, we adopt the usual grid search that maximizes the correlation between vertical rotation rate and transverse acceleration since it is the most used in all literature and provides the best visual explanation of the process. The angular step used for the grid search processing of one degree and the time window that is sliding with a 30% of overlap is one second long; no tapering is applied to the traces to avoid high correlation artifacts at the edges of the seismic traces. For BAZ analysis, the seismic traces are filtered in the [0.05,20] Hz band. We report as an example the result of two events in Figure 2. In both the sub-figures of Figure 2, we can note a high correlation portion before the p onset that is due to the Love waves present in the secondary microseism generated by the Mediterranean Sea that is better described in [10].

The resolution of BAZ with a single seismometer relies on analyzing the projections of the P onset over the radial and vertical axes which is not always clear, leaving us then with an ambiguity. The 4C based BAZ estimation is based on the value and sign of correlation between the coordinated observables (i.e., transverse acc. and vertical rotation rate), and this provides a direct solution for the π ambiguity in the data.

Provided that we can estimate the BAZ well, we now have to find a distance. In the case of seismic arrays, the apparent velocity and backazimuth can directly be measured by observing the propagation of the seismic wavefront with array methods, independently of the local seismic velocities below the station. For a single station, the distance can be obtained from the difference in arrival time of two phases, usually P and S. If we assume a constant velocity, D is the epicentral distance for surface sources, or the hypocentral distance d for deeper sources.

Equation (2) is applicable only for the travel times picked from direct crustal phases S and P and under the hypothesis of the constant $v_{p}/v_{s}$ ratio. Pg and Sg phases are usually the first onsets for local events depending on the thickness of the crust and of the source depth. After the so-called cross-over distance, the first onset can be represented by body waves dipping in the mantle or critically refracted waves at the Moho discontinuity. As a rule of thumb, we can calculate the crossover distance as five times the crustal thickness, so, by using a value of 30 km, we get a distance of 150 km that is from two to three times larger than the distance of the events studied in this work.

The picking technique for Pg and Sg onsets used to obtain the distance D is described in the following.

**Figure 3:**
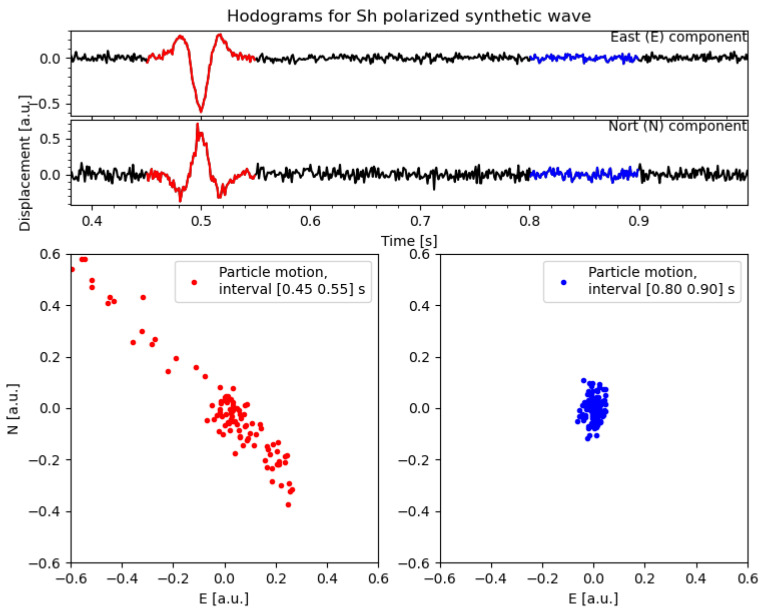
Synthetic SH wave decomposed on North and East direction (**top** panel) and the relative hodograms in different frequency bands (**bottom** panels).

The method adopted is based on the polarization properties of the wavefield, which are best illustrated by hodograms: these can be obtained by taking all possible pairs of channels in a station and then, plotting the corresponding time-series against each other, we show a synthetic example in Figure 3.

If we focus on a time window enclosing an event (that is, the seismic phase), we can see the characteristic patterns forming, as a direct consequence of the ground particle motions. These patterns are strongly dependent on the type of seismic phase and other features of the wave-front, such as amplitude and incoming direction. On the other hand, when no (strong) signal is present, noise will prevail. If such noise is not correlated among the channels, no strong patterns will emerge from the hodograms. This illustrates, quite vividly, that, by exploiting the polarization of the wave field, we can easily recognize an event in a time-window. By sliding such a time-window along the seismic recordings, we can detect the seismic phases and pick their arrival time. We will adopt a method to pick SH phases automatically.

A way to detect polarization is by studying the covariance matrix between X,Y,Z and Ω: these denote the East, North, Vertical acceleration, and vertical rotation rate components, respectively. These components should have consistent units of measurement: therefore, if Ω is in rad/s, X,Y,Z are in units of acceleration divided by a factor $\beta_{r}$ (with units [m/s]). The exact value of this factor does not matter, but should be close to the real shear wave velocity. The covariance matrix is:

(4) $$\frac{1}{N}\sum_{i=1}^{N}\left[\begin{array}{cccc} X_{i}^{2} & X_{i}Y_{i} & X_{i}Z_{i} & X_{i}\Omega_{i}\\ Y_{i}X_{i} & Y_{i}^{2} & Y_{i}Z_{i} & Y_{i}\Omega_{i}\\ Z_{i}X_{i} & Z_{i}Y_{i} & Z_{i}^{2} & Z_{i}\Omega_{i}\\ \Omega_{i}X_{i} & \Omega_{i}Y_{i} & \Omega_{i}Z_{i} & \Omega_{i}^{2} \end{array}\right]$$

where *N* is the length of the time window in samples, identified by the subscript *i*. Let us denote the eigenvalues for this matrix as $\lambda_{n}$: as a convention, we sort the eigenvalues in decreasing order. When the noise is predominant, the matrix has the maximum rank: in fact, if noise is not correlated, all the columns are independent. In such conditions, all eigenvalues are comparable. However, when a polarized event is caught in the time-window, the columns become linearly dependent and a drop in the rank occurs. In other words, the matrix becomes singular. As a consequence, there is only one non-zero eigenvalue: $\lambda_{1}>>\lambda_{2}≃\lambda_{3}≃\lambda_{4}$. Therefore, an arrival would be marked by a sudden increase in the principal eigenvalue. The eigenvector linked to $\lambda_{1}$ (henceforth, the principal eigenvector) will point in the direction of polarization. A detailed model of polarization is given in [14]. A case worth mentioning is when multiple independent arrivals coexist in the same window. For example, let us consider two. In this case, there are two non-null eigenvalues instead of one and there is no pure polarization. The key parameter of a polarization analysis is N. The length of the time window is a compromise between two requirements: Signal-to-Noise Ratio (SNR) and avoidance of coherent noise. A longer time window enhances the SNR (when the noise is incoherent) but also increases the risk of catching two independent arrivals. The recommended minimum value for the time-window is then half the dominant period of the target signal. In our analysis, a sudden increase in the principal eigenvalue is taken to mean the arrival of a seismic phase. One point to highlight is that we consciously chose to make minimal fine-tuning on N. The purpose was to keep the system as automatic as possible: this was a priority, given the number of events to analyze.

**Figure 4:**
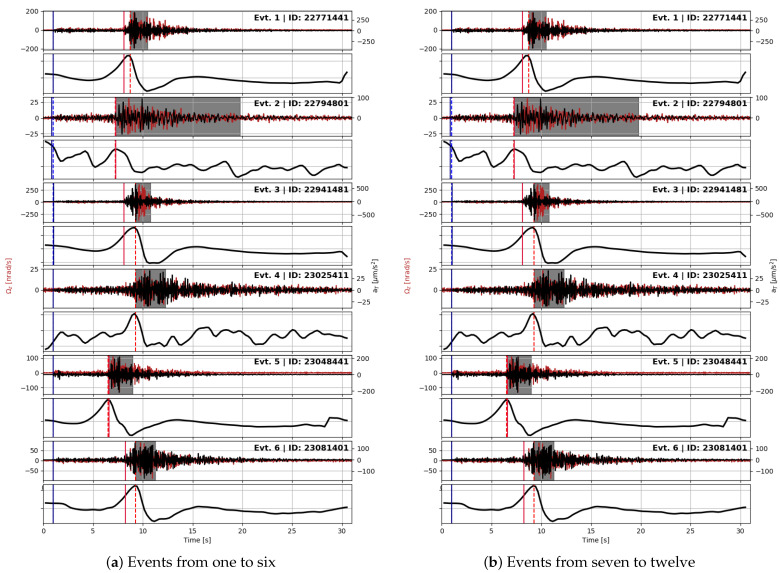
The analyzed events, from top: odd panels are a superposition of transverse acceleration (in black) and vertical rotation rate (in red), even panels the time derivative of the principal eigenvalue used for Sh waves phase picking. The latter is normalized with respect to its maximum value. Vertical lines indicate arrival times (P waves in blue, Sh waves in red) as picked by the routine used in this study (dashed lines) and published by the INGV (continuous lines). See explanation in the text for polarization analysis.

**Figure 5:**
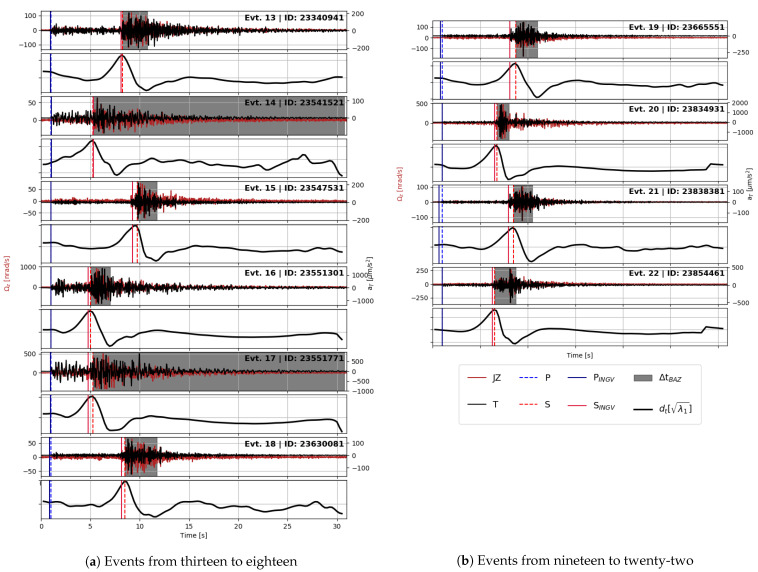
The analyzed events, from top: odd panels are a superposition of transverse acceleration (in black) and vertical rotation rate (in red), even panels the time derivative of the principal eigenvalue used for Sh waves phase picking. The latter is normalized with respect to its maximum value. Vertical lines indicate arrival times (P waves in blue, Sh waves in red) as picked by the routine used in this study (dashed lines) and published by the INGV (continuous lines). See explanation in the text for polarization analysis.

As a consequence, it was not possible to select N to minimize overlapping arrivals for every event. This means that, in some instances, two significant eigenvalues may coexist. However, this is not a problem because, in our case, the priority is to pick arrival times and not extract the wave parameters. Such an analysis will be carried out only for those events that exhibit pure polarization. A band-pass filter is also performed before the polarization analysis, to minimize coherent background noise like the secondary microseism. The band chosen is [1,20] Hz. The picking process is obtained by a Python Obspy code [23,24]. The Pg phase is picked with an autoregressive method included in Obspy from [25]. The picking of the SHg phase is based on the principle described above. We choose N=50, corresponding to 0.5 s that is half the maximum period allowed by the band-pass filter. This value is in compliance with the recommendation stated earlier, even with the conservative assumption that the dominant frequency of all examined signals corresponds to the lower cut of the filter. Before analysis, the principal eigenvalue $\lambda_{1}$ is pre-processed in two steps. First, the square root $\sqrt{\lambda_{1}}$ is taken in order to ‘compress’ the signal and enhance small variations, as suggested in [26]. Then, we compute the time derivative of this quantity, $d_{t}\sqrt{\lambda_{1}}$. Finally, the SHg phase is picked by taking the time when $d_{t}\sqrt{\lambda_{1}}$ reaches the peak. The time window used for the BAZ identification is the one spanning from the eigenvalue maximum to its minimum as shown, e.g., in Figure 4 and Figure 5. In this way, we expect to identify only the shear transverse onset, i.e., the Sg phase polarized horizontally.

## 4. Results

We collected twenty-two local earthquakes from July 2019 to February 2020 whose magnitudes are ranging from ML 2.5 to ML 3.8 and distances from 23 to 59 km from the GIGS station (see Table 1).

**Figure 6:**
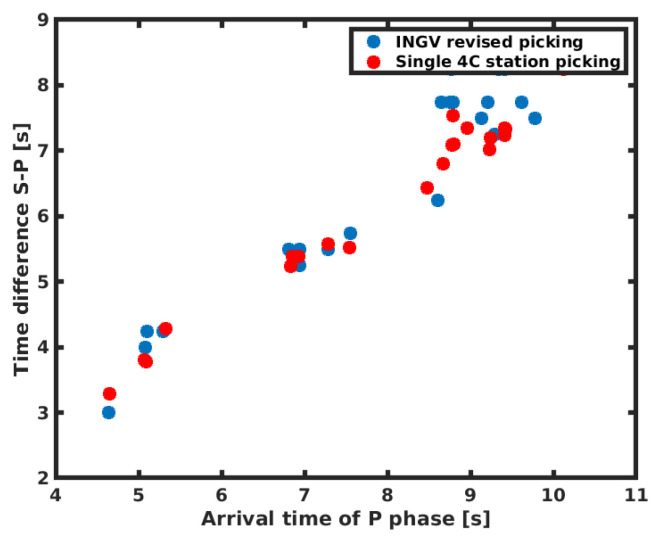
Wadati plot for the collected events for INGV bulletin picking and for our picking method.

For all these events, we applied our picking routine, and we obtained the results shown in Table 2. We provide a comparison between the automatic picking routine with the picking values published by the INGV after their final revision in Figure 4 and Figure 5b. The results of the picking routine are represented by dashed lines, while the INGV values by continuous lines. As an independent verification, we plot the arrival time of the p onset from source time versus S to P time difference, the so-called Wadati plot. We plot these variables for both the INGV picking and for our method in Figure 6.

After a linear regression fit, we could extract the $V_{p}/V_{s}$ values that are of $v_{p}/V_{s}=1.93\pm 0.06$ for INGV picking and of $v_{p}/V_{s}=1.84\pm 0.03$ for our picking method. We performed this analysis using a Wadati diagram to obtain an independent check of the observed arrival times. No points not fitting with the linear relationship were found, suggesting no misreadings or wrong phase identification (Figure 1).

After checking the good agreement of picking times, we performed a linear regression between the hypocentral distance provided by the INGV and the time difference $\Delta T_{ps}$ obtained by our method. The regression method used suggests a linear model where the angular coefficient represents the $\frac{v_{p}v_{s}}{v_{p}-v_{s}}$ value.

**Figure 7:**
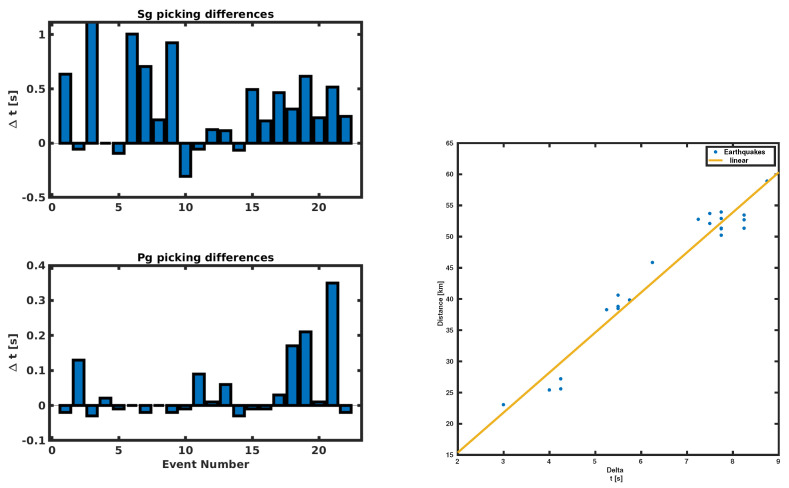
Right panel: The picked time differences compared with the hypocentral distance provided by the INGV localization and the linear regression. Left panel, on top, the time difference in [s] from INGV to AR-pick Pg onset. The bottom panel represents the time difference in [s] from INGV to 4C polarization Sg onset picking.

The value we get from the fit is 7±0.5; if we assume a Poisson solid where $v_{p}=V_{s}\sqrt{3}$, we get reasonable values of $v_{p}/v_{s}=1.73$, vp=5.1 and $v_{s}=2.9$. The values obtained are comparable to others reported in literature e.g., in [27,28]. The results of his analysis are reported in Figure 7.

**Figure 8:**
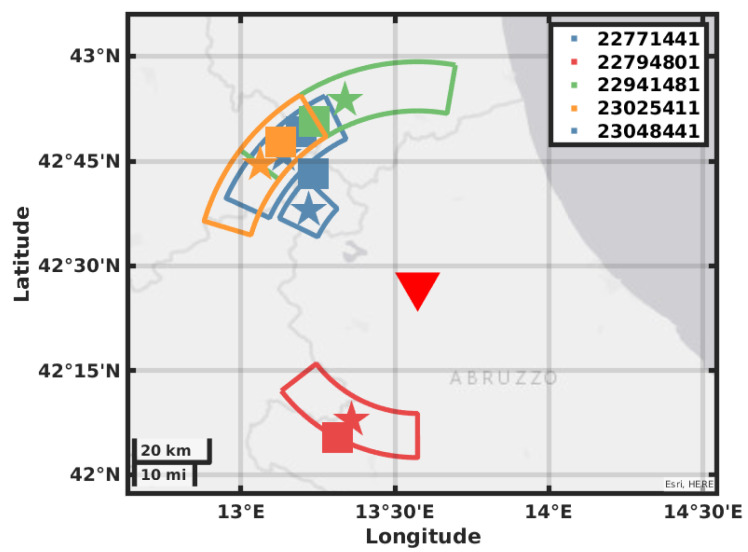
Map showing the GIGS station location, the earthquakes located by the INGV in solid squares, the earthquakes located using the GIGS station, and the relative location error in polar coordinates.

**Figure 9:**
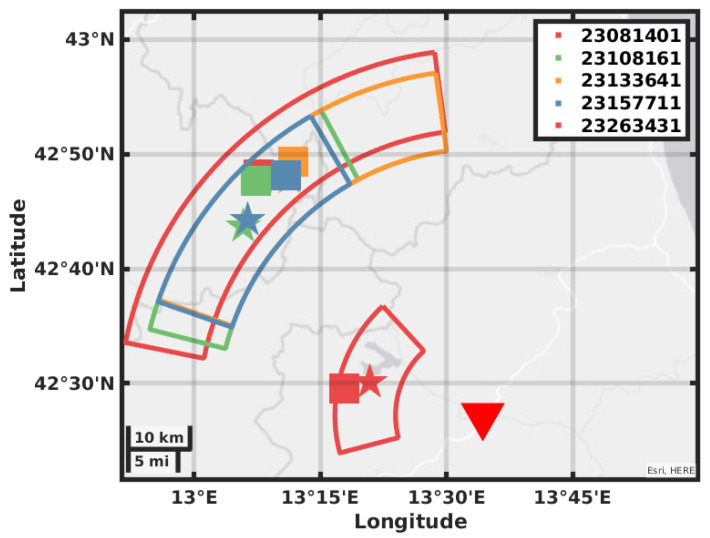
Map showing the GIGS station location, the earthquakes located by the INGV in solid squares, the earthquakes located using the GIGS station and the relative location error in polar coordinates.

**Figure 10:**
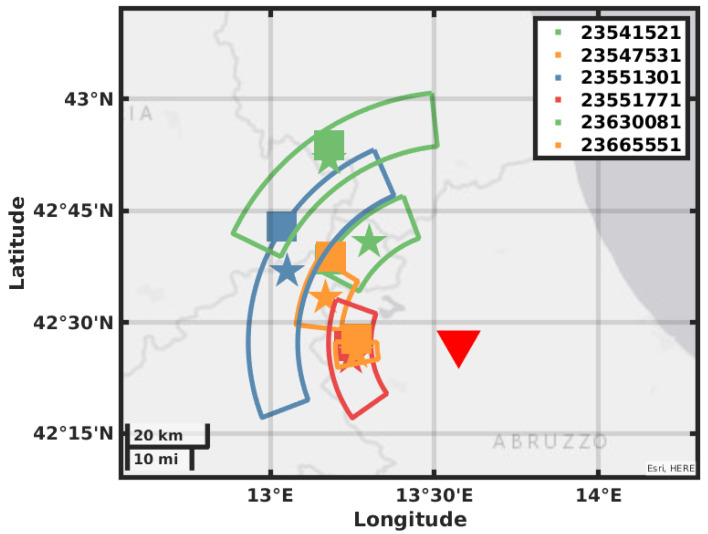
Map showing the GIGS station location, the earthquakes located by the INGV in solid squares, the earthquakes located using the GIGS station and the relative location error in polar coordinates.

**Figure 11:**
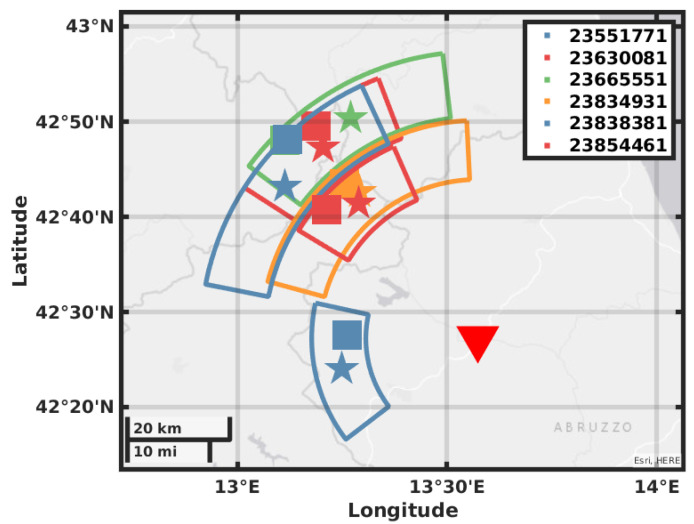
Map showing the GIGS station location, the earthquakes located by the INGV in solid squares, the earthquakes located using the GIGS station and the relative location error in polar coordinates.

The value of BAZ is obtained by picking the maxima of ZLCC in the time window obtained by the eigenvalues analysis; the values are reported in Table 3 in the mean value of the picked values and the error is the standard deviation. In Figure 8, Figure 9, Figure 10 and Figure 11, we plot on a map the position of the station GIGS, the events localized by the INGV service, and the events located with the above method. The error on distance is obtained after error propagation in Equation (2) where two errors contribute. The first one is a time picking error that we estimate to be of half a second. The second one is the error associated with the linear regression for the estimation of the factor $\frac{v_{p}v_{s}}{v_{p}-v_{s}}$. An alternative method can be to use the value of the apparent velocity for Sg phase reported by our previous study in [11] of 2.7 km/s to calculate the distance D, being then independent of the INGV earthquake monitoring service. In fact, using this estimation provided by the instrument itself and a theoretical ratio from $v_{p}$ to $v_{s}$ of $1/\sqrt{3}$, we could still locate the earthquakes but with a larger error in distance, i.e., the error provided by the $v_{s}$ estimation in [11].

Since we have the possibility to compare our location to the one obtained with an average of more than two hundred stations used by INGV, we prefer to use a “learning” approach for our 4C station with the target to automate this procedure at the station and perform a cluster analysis on the differences of P to S arrivals, possibly highlighting the constant distance and BAZ dependent biases in our estimations.

Our location gives a result in polar coordinates; the error areas therefore assume the shape of a circular sector covering the possible epicenter location. The location error of the INGV is roughly five times smaller than the size of the square that we use as a marker; this has been enlarged for plotting reasons. As we can see for most of the events, the distance that we obtain with the relative error contains the location in terms of radius. The direction estimation shows a larger error with a basically correct mean value; this may be due to the method used for error estimation or by geophysical reasons like a large scattering near the receiver.

## 5. Conclusions

The upgrades in the acquisition system of Gingerino station permitted to record and access in a simpler way the precious seismic data recorded by the 4C seismic station. The selected events were analyzed with the purpose of testing a way to implement in the station hardware an automatic processing that can lead to a first single station raw earthquake location for short epicentral distances. The method is based on the classical BAZ identification by correlation with the canonical observable vertical rotation rate–transverse acceleration. The automation process involves the automatic picking of crustal Pg and Sg phases. This was implemented in a code where the more complex Sg phase picking is made simpler and stable by exploiting the polarization properties of the wave field. The result obtained suggests values of $v_{p}$ and $v_{s}$ consistent with literature and with the estimation of $v_{s}$ obtained previous works on this station. The largest part of the events are located in an area that includes the more accurate and precise location of the INGV. By the time of writing this paper, more than 100 earthquakes of magnitude larger than 2.0 occurred from February 2020 in a range of 50 km. The development of an automatic processing routine will permit a more detailed analysis and a deeper learning process for our station with the perspective of future applications in different and less instrumented environments.

## Figures and Tables

**Table 1:** List of the events analyzed in this study.

ev_ID	Time	Lat	Lon	Dist. [km]	Depth [km]	Mag (ML)
22771441	2019-07-30 07:53:43.230	42.821	13.1952	51.2556652901561	9.8	2.9
22794801	2019-08-03 08:39:50.240	42.0882	13.3133	45.8580189202651	9.7	2.6
22941481	2019-08-26 07:23:37.800	42.8433	13.2367	51.358086956751	10.9	2.8
23025411	2019-09-05 04:03:56.220	42.7957	13.1277	52.698075225214	9.1	2.6
23048441	2019-09-08 16:28:13.460	42.7203	13.2345	40.6142950290862	10	2.5
23081401	2019-09-14 10:18:13.420	42.8047	13.1272	53.4519454861873	9.6	2.8
23108161	2019-09-18 19:36:43.750	42.7952	13.1232	52.9133199257223	9.4	3.2
23133641	2019-09-22 01:28:40.010	42.8225	13.1958	51.3591488287935	9.6	3
23157711	2019-09-25 10:41:44.590	42.8022	13.183	50.2341404536896	10.1	2.5
23263431	2019-10-12 12:29:55.330	42.4923	13.2968	23.0538380588901	9.9	3.1
23271311	2019-10-14 06:28:51.610	42.6413	13.1813	38.2932402297496	10.8	2.5
23272401	2019-10-14 09:02:38.400	42.6467	13.1833	38.4874482403291	9.4	2.6
23340941	2019-10-28 07:14:39.080	42.7148	13.035	52.7755051887485	9.8	3
23541521	2019-12-05 16:08:25.890	42.4468	13.2413	27.2104978980552	11.3	2.5
23547531	2019-12-07 05:31:02.320	42.8967	13.1787	58.9109750493387	9.6	2.6
23551301	2019-12-07 21:55:38.130	42.4628	13.2633	25.4155919945919	13.7	3.8
23551771	2019-12-07 22:16:54.620	42.4602	13.261	25.5941756227566	10.6	3.4
23630081	2019-12-20 23:19:58.450	42.8263	13.1873	52.1148418310692	10.6	2.5
23665551	2019-12-25 23:26:37.320	42.8007	13.1127	53.9493563219997	9	2.6
23834931	2020-01-28 16:37:34.640	42.7243	13.2548	39.8346087439138	8.2	3.3
23838381	2020-01-29 01:24:59.840	42.8028	13.1197	53.7196183595932	9.8	2.5
23854461	2020-02-01 15:09:49.410	42.6793	13.2117	38.8191854652558	10.1	2.8

**Table 2:** Comparison between p and s onset picking, INGV seismic bulletin values compared to our method.

ev_ID	P DATE	P_INGV	P_picked	S_INGV	S_picked
22771441	30-Jul-2019	07:53:52:010	07:53:51:990	07:53:59:100	07:53:59:735
22794801	03-Aug-2019	08:39:58:720	08:39:58:850	08:40:05:150	08:40:05:095
22941481	26-Aug-2019	07:23:46:600	07:23:46:570	07:23:53:700	07:23:54:815
23025411	05-Sep-2019	04:04:05:540	04:04:05:561	08:47:04:561	04:04:13:806
23048441	08-Sep-2019	16:28:20:750	16:28:20:740	16:28:26:330	16:28:26:235
23081401	14-Sep-2019	10:18:22:830	10:18:22:830	10:18:30:070	10:18:31:075
23108161	18-Sep-2019	19:36:52:980	19:36:52:960	19:37:00:000	19:37:00:705
23133641	22-Sep-2019	01:28:48:800	01:28:48:800	01:28:56:330	01:28:56:545
23157711	25-Sep-2019	10:41:53:260	10:41:53:240	10:42:00:060	10:42:00:985
23263431	12-Oct-2019	12:29:59:980	12:29:59:970	12:30:03:270	12:30:02:965
23271311	14-Oct-2019	06:28:58:460	06:28:58:550	06:29:03:850	06:29:03:795
23272401	14-Oct-2019	09:02:45:330	09:02:45:340	09:02:50:710	09:02:50:835
23340941	28-Oct-2019	07:14:48:320	07:14:48:380	07:14:55:510	07:14:55:625
23541521	05-Dec-2019	16:08:31:220	16:08:31:190	16:08:35:500	16:08:35:435
23547531	07-Dec-2019	05:31:12:440	05:31:12:430	05:31:20:680	05:31:21:175
23551301	07-Dec-2019	21:55:43:220	21:55:43:210	21:55:47:000	21:55:47:205
23551771	07-Dec-2019	22:16:59:690	22:16:59:720	22:17:03:500	22:17:03:965
23630081	20-Dec-2019	23:20:07:410	23:20:07:580	23:20:14:760	23:20:15:075
23665551	25-Dec-2019	23:26:46:730	23:26:46:940	23:26:54:070	23:26:54:685
23834931	28-Jan-2020	16:37:42:180	16:37:42:190	16:37:47:700	16:37:47:935
23838381	29-Jan-2020	01:25:09:270	01:25:09:620	01:25:16:600	01:25:17:115
23854461	01-Feb-2020	15:09:56:240	15:09:56:220	15:10:01:470	15:10:01:715

**Table 3:** Results and comparison for distance and backazimuth estimation.

Event ID	D [km]	HypD [km]	EST. D [km]	Δ D	BAZ [deg]	EST. BAZ [deg]	Δ BAZ
22771441	51.2556652901561	52.1841280883039	49.5679950714111	6	323.065506757991	328	28
22794801	45.8580189202651	46.8726775349072	39.9680471420288	5	207.841005121603	185	18
22941481	51.358086956751	52.502029445129	52.7679777145386	6	327.750507363209	330	3
23025411	52.698075225214	53.4780060627013	52.7679777145386	6	316.432971160146	312	13
23048441	40.6142950290862	41.8272753201742	35.1680088043213	5	317.115124375038	352	37
23081401	53.4519454861873	54.3071862303538	52.7679777145386	6	317.144876972473	303	40
23108161	52.9133199257223	53.7417847262429	49.5679950714111	6	316.104623798978	323	9
23133641	51.3591488287935	52.2486570967921	49.5679950714111	6	323.221471377853	330	7
23157711	50.2341404536896	51.23942688127	49.5679950714111	6	320.719262138433	319	24
23263431	23.0538380588901	25.089628320195	19.1680312156677	4	280.972959907815	295	24
23271311	38.2932402297496	39.7870864388599	33.5679531097412	5	303.245455188636	309	2
23272401	38.4874482403291	39.6187288040898	35.1680088043213	5	304.127897489016	311	26
23340941	52.7755051887485	53.6776857542091	46.3680124282837	6	303.634132116785	308	20
23541521	27.2104978980552	29.4635570809104	27.1679878234863	5	268.620966580245	275	6
23547531	58.9109750493387	59.688047222738	55.9680247306824	6	326.975839950607	329	2
23551301	25.4155919945919	28.8728647078111	25.5679965019226	5	272.519915351149	281	1
23551771	25.5941756227566	27.7023794250333	27.1679878234863	5	271.856197317918	261	1
23630081	52.1148418310692	53.181921167605	47.9680037498474	6	322.892905049481	293	24
23665551	53.9493563219997	54.6949087901067	49.5679950714111	6	315.905232456936	319	2
23834931	39.8346087439138	40.6698420673193	36.768000125885	5	319.294223601461	332	7
23838381	53.7196183595932	54.606202914141	47.9680037498474	6	316.51468132474	315	7
23854461	38.8191854652558	40.1115838653366	35.1680088043213	5	310.493568388689	313	13

## Data Availability

The data used for this analysis are available on EIDA at IV.GIGS.*.

## Abbreviations

The following abbreviations are used in this manuscript:

RLG	Ring laser gyroscope
BAZ	Back azimuth
INGV	Istituto Nazionale di Geofisica e Vulcanologia
INFN	Istituto Nazionale di Fisica Nucleare
ZLCC	Zero lag correlation coefficient
